# Using Morphological Data in Language Modeling for Serbian Large Vocabulary Speech Recognition

**DOI:** 10.1155/2019/5072918

**Published:** 2019-03-03

**Authors:** Edvin Pakoci, Branislav Popović, Darko Pekar

**Affiliations:** ^1^Department for Power, Electronic and Telecommunication Engineering, Faculty of Technical Sciences, University of Novi Sad, 21000 Novi Sad, Serbia; ^2^AlfaNum Speech Technologies, 21000 Novi Sad, Serbia; ^3^Department for Music Production and Sound Design, Academy of Arts, Alfa BK University, 11000 Belgrade, Serbia

## Abstract

Serbian is in a group of highly inflective and morphologically rich languages that use a lot of different word suffixes to express different grammatical, syntactic, or semantic features. This kind of behaviour usually produces a lot of recognition errors, especially in large vocabulary systems—even when, due to good acoustical matching, the correct lemma is predicted by the automatic speech recognition system, often a wrong word ending occurs, which is nevertheless counted as an error. This effect is larger for contexts not present in the language model training corpus. In this manuscript, an approach which takes into account different morphological categories of words for language modeling is examined, and the benefits in terms of word error rates and perplexities are presented. These categories include word type, word case, grammatical number, and gender, and they were all assigned to words in the system vocabulary, where applicable. These additional word features helped to produce significant improvements in relation to the baseline system, both for n-gram-based and neural network-based language models. The proposed system can help overcome a lot of tedious errors in a large vocabulary system, for example, for dictation, both for Serbian and for other languages with similar characteristics.

## 1. Introduction

There are two main components in any contemporary automatic speech recognition (ASR) system. The first is the acoustic model (AM), which describes acoustical characteristics of different speech components (most often context-dependent phonemes) for a single speaker (in speaker-dependent or speaker-adapted systems) or multiple speakers (in speaker-independent systems). The other component, on which this manuscript will be focused, is the language model (LM), which describes the vocabulary and sentence forming rules of the language or speech domain in question. The language model is used to provide the speech recognizer with allowed word sequences in limited-vocabulary and grammar-based environments, as well as to help the acoustic model to decide on the correct word sequence by introducing costs for all different sequences (i.e., language model costs, or scores), where the more likely sequences will have a lesser cost (a better score). In a lot of applications, a well-trained language model can even overcome certain flaws in the acoustic model, by eliminating unnatural, unlikely word sequences from the list of recognition result possibilities. It has been shown that language models have the capability to become very close to human language understanding [1].

For a long time, the best language models in existence were statistical models based on *n*-grams—frequencies or probabilities of individual word sequences up to and including length *n* [2]. These LMs proved to be highly effective for an array of applications, even though they had several known problems, e.g., data sparsity (smoothing requirement [3]) and modeling of longer contexts (more than *n* words long). Recently, approaches based on recurrent neural networks (RNNs) have been proposed to overcome *n*-gram issues without raising the implementation difficulty and computational complexity too much. They have shown their superiority in relation to *n*-grams [4], but still they are computationally more demanding, and that usually results in a lot longer training duration.

For the Serbian language, in the past couple of years, several variants of RNN-based LMs (RNNLMs) as language models were examined and compared [5]. All of them produced big improvements over the baseline *n*-gram system, while the best approach seemed to be the TensorFlow-based LSTM-RNNLM (long short-term memory-based RNNLM) approach with pruned lattice rescoring, both in the resulting word error rates (WERs) and training duration. Unfortunately, a number of problems from the *n*-gram system seemed to remain. The biggest ones were errors where the lemma was correct, but the word ending was wrong, which resulted in a very low character error rate (CER) in comparison to the actual WER. The source of this issue was deemed to be high language inflectivity of Serbian—the same basic word form (lemma) can have a lot of different word suffixes describing different grammatical or syntactic roles (Table 1). In Serbian grammar, there are seven word cases (nominative, genitive, dative, accusative, vocative, instrumental, and locative), which apply to all nouns and most adjectives, as well as some pronouns and numerals, two grammatical numbers (singular and plural), and three grammatical genders (masculine, feminine, and neuter). Grammatical numbers and genders apply to most verbs as well. Cases, numbers, and genders do not apply to invariable words (prepositions, adverbs, conjunctions, particles, and exclamations), even though certain prepositions are always followed by certain cases.

In this manuscript, incorporation of the mentioned morphological features into both *n*-gram based and RNN-based language models for Serbian is examined, and the obtained results are presented on the largest Serbian audio database for acoustic modeling, as well as all the currently available textual materials in Serbian for language model training.

The following sections will describe relevant previous work, details of the available resources, training methods, the experimental setup, and the results, followed by conclusions.

## 2. Relevant Previous Work

There were several approaches for incorporation of morphology knowledge into speech recognition systems for other languages, and most of them require some sort of a parser (word decomposer) to determine significant morphological units (morphemes, affixes, etc.) to represent lexical items and word classes, and then that information is used to provide additional constraints to the decoder (in combination or instead of regular words in the conventional approach). A lot of morphologically rich languages face similar issues [6–10].

Another approach is using factored language models (FLMs) [11], which explicitly model relationships between morphological and lexical items in a single language model, and a generalised back-off procedure is used during training to improve the robustness of the resulting FLM during decoding, especially for rarely seen words and *n*-grams. In the approach in this manuscript, additional morphological information about all the words in the textual corpus for LM training is explicitly embedded into the words themselves, and the LM training is performed on this modified vocabulary. Given the fact that Serbian is a fusional language, which are distinguished from agglutinative languages by their tendency to use a single inflectional morpheme to denote multiple grammatical, syntactic, or semantic features (and that has been a problem for some morphology models [8]), and the planned usage of the ASR system in question in large but relatively finite vocabulary environments (specific domains with a lot of expected words and phrases), such an approach is justifiable, but future research should look into the possibilities of creating open vocabulary systems as well [12, 13].

## 3. Materials and Methods

### 3.1. Audio Database

For all the experiments, the recently expanded speech database for Serbian was used. This database consists of three smaller parts (Table 2). The first part contains audio book recordings, recorded in studio environment by professional speakers. A large part of this database was already mentioned in previous papers [14], but lately it has been expanded by several new audio books. This database part is responsible for 168 hours of data in total, out of which about 140 hours is pure speech (the rest is silence). There are 32 different male and 64 different female recognized speakers (with a slight possibility that a few of them are actually the same speaker), but male speakers had a lot more material by speaker on average. The original data for each speaker were further separated into chunks of 30–35 minutes at most, and all chunks except the first were modified by a carefully chosen combination of speech tempo or pitch changes, basically producing new, mutually distinct subspeakers. The purpose of this procedure was to equalize the amount of material per speaker, as in the original data some speakers have several hours of speech, while others have half an hour or even less. In this way, the trained acoustic models should not be biased towards those speakers with a lot of material. The described procedure resulted in 398 distinct subspeakers. The second part of the database contains radio talk show recordings, separated by speaker. This part totals 179 hours of data, 150 of which are nonsilence, and there are 21 male and 14 female speakers in total, again with a lot more material for males. Speaker equalization (in the same manner as above) was also performed here to produce 420 subspeakers. These recordings contain mostly more spontaneous speech, with a lot more background noise, mispronounced words, etc., but are crucial for better modeling of conversational speech. The final database part is the so-called Serbian “mobile” speech database, also mentioned in previous papers [15], and consists of mobile phone recordings of read commands, questions, numbers, dates, names, locations, spellings, and other inquiry-based utterances, like those to be expected in an interaction with a voice assistant type application on a smartphone. These are also more freely spoken, but the utterances are a lot shorter than those in previous database parts, the vocabulary is very domain-oriented and relatively small, and the material is already evenly distributed among speakers. This part contains 61 hours of material, out of which 41 are pure speech, and there are 169 male and 181 female distinct speakers. All audio data for acoustic model training were sampled at 16 kHz, 16 bits per sample, mono PCM.

In addition to this, for testing purposes, 29 hours of material was extracted in total (between 5% and 10% from all database parts), 23 of which is speech, from 81 total test subspeakers. All subspeakers used in the test set were completely used for testing (i.e., excluded completely from training) to avoid biased test results.

### 3.2. Textual Corpus

All the language models that are going to be mentioned were trained on the same textual corpus. The largest part of it are texts previously collected for Serbian language model training [5, 15], divided into segments corresponding to different functional styles—the largest journalistic corpus, followed by literary, administrative, scientific, popular-scientific, and conversational segments. The whole corpus was used in an attempt to cover as much variability as possible, as it has been shown that sentence structures in different functional styles can differ significantly [16]. Additionally, the transcriptions of the training part of the audio data for acoustic modeling were appended to the existing corpus. In total, there are about 1.4 million sentences and 26 million words. Out of these, 20000 sentences were used only for evaluation (the development, or “dev” set), while the rest were used in the language model training procedure (Table 3).

### 3.3. Training Method—Acoustic Model

The used acoustic models were subsampled time-delay neural networks (TDNNs), which are trained using cross-entropy training within the so-called “chain” training method [17]. For this purpose, the Kaldi speech recognition toolkit [18] was used. The trained neural network is 9 layers deep, with 625 neurons per layer. The initial layers (1–5) were spliced in a {−1, 0, 1} manner (they see 3 consecutive frames), while {−3, 0, 3} splicing was used for the most hidden layers (layers 5–9; they also see 3 frames, but separated by 3 frames from each other). Using this configuration, the most hidden layers need to be evaluated only every 3 frames. No artificial data expansion was used for these experiments. The training was performed in 5 epochs (145 iterations based on the amount of data). Alignments for the deep neural network (DNN) training were provided by a previously trained speaker-adaptive HMM-GMM (hidden Markov model—Gaussian mixture model) system [19] with 3500 states and 35000 Gaussians. Acoustic features used for DNN training were 40 high-resolution MFCC features (Mel-frequency cepstral coefficients), alongside their first- and second-order derivatives, as well as 3 pitch-based features—weighted log-pitch, delta-log-pitch, and warped normalized cross-correlation function (NCCF) value (which is originally between −1 and 1, and higher for voiced frames), and their derivatives, producing a 129-dimensional feature vector, which is a configuration already used in other experiments [5, 15, 17]. The context dependency tree used for the “chain” training with its special model topology that allows a subsampling factor of 3 had 2000 leaves (output states). The effective learning rate was in the range from 0.001 (initial) to 0.0001 (final).

### 3.4. Training Method—Language Models

The referent *n*-gram language model is a 3-gram model trained on the described textual data using the SRILM toolkit [20], with Kneser-Ney smoothing and previously optimized pruning cut-off parameter of 10^−7^ [15]. The vocabulary for the LM was chosen in such a way to include all different words from the acoustic training data transcriptions, plus all other words that are mentioned at least 3 times in the whole textual corpus. Additionally, previously unseen words from the test dataset transcriptions were also added into the vocabulary, so there were no actual out-of-vocabulary (OOV) words, but these transcriptions were not used in probability estimation for the LM. Still, it should be acknowledged that adding OOV words to the LM training vocabulary can affect the recognition accuracy of the ASR system. This approach was related to the planned use of this system (relatively finite-vocabulary domains) and the fact that the experimental results needed to demonstrate the expected WERs in such conditions. Moreover, a similar approach was used previously as well [5, 15], but future research and experiments should measure the WER without adding all the OOV words from the test dataset into the vocabulary and even consider an open vocabulary language model capable of learning new words. The procedure used here resulted in 249809 total words (unigrams), while there were also 1.87 million bigrams and 551 thousand trigrams with the given parameters. The test data perplexity was calculated to be around 634.0.

The RNN-based language model was trained using Kaldi-RNNLM [21], an extension to the Kaldi toolkit, which supports RNN-based language modeling within the Kaldi framework and weighted finite state transducer- (WFST-) based decoding. This method involves subword features; more precisely, letter *n*-gram counts for better prediction of rare words, as well as augmented features such as scaled word unigram log-probability and word length, the former of which is used for better out-of-domain results. Kaldi-RNNLM also shares the input and output embeddings for the neural network based on work given in [22], which alongside subword features can produce good results on very large vocabularies without having data sparsity issues (which is otherwise usually combatted by using shortlists during LM training). Finally, each of the most frequent *N* words receives an additional feature, so the top words end up having a one-hot representation in addition to their letter *n*-gram counts vector and the two augmented features (Table 4).

The baseline RNNLM is a 4-layer combined TDNN plus fast LSTMP (LSTM projected [23]) network, with an embedding dimension of 1024 and both recurrent and nonrecurrent projection dimension of 256. The number of most frequent words to receive a special feature is 97636 (calculated to be up to 100000, but to draw a line under a group of words with the same count in the input data). Letter 2-grams and 3-grams are utilized, the minimum frequency of any letter *n*-gram to be considered a feature was 0.0001, and the training was run for 30 epochs (180 iterations based on input data), with the possibility for the best iteration to be before the last one (best iteration is calculated based on the objective function value on the “dev” dataset previously mentioned in the textual corpus section). For RNNLM rescoring, the pruned lattice rescoring method was used [24] with a 4-gram approximation to prevent lattice explosion and a RNNLM interpolation weight of 0.8 (previously determined to be optimal). The baseline perplexity with this RNNLM on the given test set was calculated using Kaldi tools to be about 119.0.

The approach to incorporation of morphological information into the language model for Serbian in this manuscript is to explicitly embed that information into the words themselves, thus modifying the vocabulary of the ASR system. In order to figure out all the different morphological categories for each word in the input textual corpus sentences, a part-of-speech (POS) tagging tool for Serbian [25] was used, alongside the Serbian morphologic dictionary [26]. Previously, morphological clustering of words into classes using a part of the Serbian textual corpus was examined, where the relevant features were defined for each word type (case, number, and gender, as briefly mentioned in the introduction section of this manuscript, alongside subtype, e.g., proper, common, or abstract for nouns and degree of comparison for adjectives) [27]. Not all the additional features are available for all word types, even within a certain type some words do not behave like others, e.g., there are some invariable adjectives. For the following experiments, word type and case, alongside grammatical number and gender, are chosen as additional word features, and for the final one, the corresponding lemma was taken into account as well.

The POS tagging tool and an additional postprocessing tool were used to convert all input textual data for LM training into sentences with tagged words, i.e., words with one or more delimited suffixes for each word denoting its determined type, case, number, and gender, where applicable. Alongside the ten word types in Serbian, two additional types were introduced—abbreviation and isolated letter (e.g., used when spelling something), since they do not really belong in any other category. Some words were marked by the POS tagger as of unknown type (e.g., badly pronounced words which were written as such in transcriptions or words with typographical errors), so they were not assigned any other morphological features. The POS tagger, with the help of the morphologic dictionary, could distinguish six different cases, as dative and locative in Serbian tend to share the same word form. A case is also assigned to certain prepositions, if they are known to always be followed by a word in a particular case in Serbian. The grammatical number and gender did not receive any special treatment; they are used as already described above (Table 5).

Using the proposed procedure, the number of different words in the LM vocabulary grew to 380747, as some words could, as expected, have different values of certain POS features (sometimes the same word form could be a different combination of case/number/gender in different sentences, even a different word type in some cases). Using the same parameters for smoothing and pruning, the new 3-gram language model has 2.2 million bigrams and 523 thousand trigrams (relatively similar to the referent 3-gram LM). This time though, the perplexity was calculated to be 378.6, which is a lot better, likely because now there is a distinction between formerly same words that could have completely unrelated functions in sentences. On the other hand, the perplexity for the new RNNLM was a bit larger than that for the referent one—147.1, which may be explained by the implicit vocabulary size increase, which had more effect here in relation to the *n*-gram case (possibly due to applied smoothing and pruning techniques there).

## 4. Results and Discussion

### 4.1. 3-Gram Results

The baseline 3-gram language model (250k words, without using morphological information) in combination with the resulting acoustic models trained with the “chain” method produced a word error rate of 8.89%. Problems with the inflectivity of Serbian can be observed when comparing that to the character error rate, which in this experiment was measured to be only 2.63%. The largest number of recognition errors happened in the radio talk show test set (12.64% WER), and the error rate for audio books was in the middle of the road (6.25% WER), while the mobile phone test set produced a very small WER of less than 1% (0.96%), just like in past experiments [15], which can be explained by the very small vocabulary (less than 4000 different words) and repeating word patterns and sentence structures for basically all speakers in this dataset, so the language model could learn to predict such sentences very well. When looking through the list of the most substituted words, on the top of it, the typical confusion between similarly sounding *i* and *je* (Eng. *and*, *be*) can be found, as well as a lot of wrong cases, grammatical genders, and numbers (*koja* instead of *koji*, *koje* or *koju*, and vice versa; Eng. *which*), but also words that have two slightly different but functionally completely equivalent forms (e.g., *kad* and *kada*, Eng. *when*), as well as several obvious typographical errors and words that are often shortened in spontaneous speech (e.g., *znači* and *‘nači*, where the starting “*z*” sound is often not pronounced at all, likewise *rekao* and *rek'o*; Eng. *so*, *told*). Some of these errors should be automatically corrected by taking morphological features into account. On the other hand, the typographical errors can only be fixed by carefully looking through all the texts by a group of text checkers.

In comparison, when applying the new 3-gram language model which differentiated POS categories of words, the WER was lowered to 6.90%, and the CER to 2.20%, which is a 22% relative improvement in WER and a 16% relative improvement in CER (Table 6). A breakdown by test database part (audio books, radio talk shows, and phone recordings) shows that the most relative improvement occurred in audio books, possibly due to professionally read texts (no unexpected or mispronounced words and sentence structures most of the time). Somewhat less improvement can be observed for radio talk shows, while a very small deterioration happened for the mobile phone database, even though the error rate is still around the 1% WER mark, probably because of more spontaneity in speech for these two test set parts and likely POS tagger mistakes and/or limitations when used on unconventional word forms encountered there (maybe even transcription errors for talk shows or erroneously recorded audio files for the mobile phone database). The total number of substitutions dropped by more than 25%. The number of wrong-POS-category errors dropped as well, and they were more spaced-out through the list of most common errors (they were more rarely seen in relation to other errors). Insertion rate dropped by 19% and deletion rate by 9%—these errors mostly included very short invariable words, and with the new LM, some occurrences of longer and variable words disappeared from the top of those error lists (Table 7).

### 4.2. RNNLM Results

The first RNNLM, without morphological features, already gave improvements across the board in comparison to both 3-gram systems. The average WER of 4.90% is a 46% relative improvement to the baseline 3-gram system and a 29% improvement to the 3-gram-POS system. The CER vas measured to be 1.61%. The biggest step forward occurred in the audio books database part again (2.77% WER), but a large step forward was made for radio shows as well (7.56% WER), and even for mobile phone recordings (0.73% WER). Looking at substitutions, insertions, and deletions, the same distribution of errors exists as for the baseline 3-gram system, there are just a lot less of them in absolute numbers.

**Figure 1:**
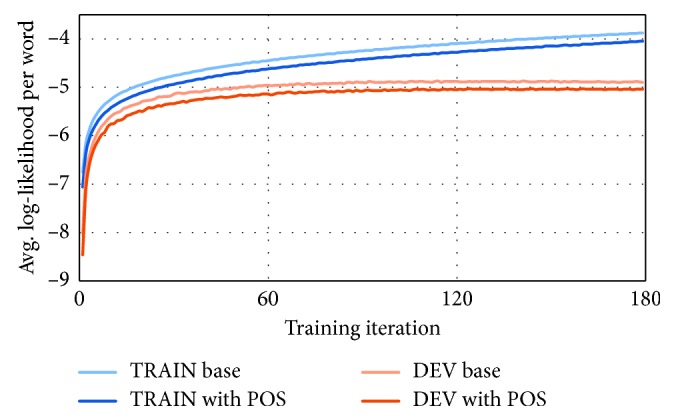
Plot of average log-likelihoods per word during training with respect to training iterations, recorded over 180 iterations in total, for both RNNLM experiments. Likelihoods for the training set (blue lines) and for the “dev” set (red lines) are separated as well.

The RNNLM system with morphological data taken into account produced further improvements in WER and CER—4.34% WER on average and 1.48% CER (Table 8). Best relative improvement was seen for audio books (21%), while radio show error rate lowering was a bit smaller (8%), and phone recordings suffered a 10% relative WER increment (the absolute error rate is still very low), just like in *n*-gram experiments, and probably for the same reasons. Likelihoods during training, both for the actual training data, and the “dev” data, show consistently slightly better values for the baseline RNNLM, probably due to the same set of reasons as for the difference in perplexities (Figure 1), and it has also been shown that a better perplexity does not necessarily mean a better WER and vice versa [28]. A better way to choose a representative “dev” set should be considered as well. The top list of errors by type, especially the substitutions list, now mostly holds errors that can be categorized as of lower significance. As mentioned before, there are a lot of either typographical errors or badly-pronounced-word errors, words with more than one equivalent similar form in regular usage, etc. The effect is even clearer than in the *n*-gram case.

The last experiment was related to the usage of lemmas, i.e., basic forms of words, as additional information for RNNLM training. Similarly to how the most frequent words had their own feature (a one-hot vector representation as a subvector of their own word features), the most frequent lemmas were also given special features, so the words whose lemmas are in this set had an additional one-hot vector as a feature, representing the lemma. The number of top lemmas was chosen to be equal to the number of top words (97k). This experiment produced the best results on the given test database so far—a WER of 4.23% and CER of 1.45%. Even though the resulting feature and word embedding matrices for the RNNLM are quite larger in this configuration (as there are a lot more individual word features), the decoding speed does not suffer (but memory consumption issues have to be prevented in this case by not using machines with insufficient memory capacity).

Further improvements can be made in several different parts of the ASR system. Firstly, the acoustic models can probably be improved a bit, both with neural network parameter optimization and with audio database augmentation (e.g., by using speech speed perturbance algorithms, or audio with artificially added noise to improve system robustness). There can be improvements in RNNLM training as well—one way is to optimize training parameters a bit more and another is to make more complex networks, but that will lead to slower decoding speeds. Finally, the textual data can be cleaned up and expanded—one way to do the cleaning is by using a simple text processor tool to fix at least the most common mistakes from the top errors lists, which can also be used as a recognition result postprocessor with the current system. Currently, there is an additional textual database in preparation for future trainings. For usage in specific domains, one type of RNNLM training texts can be used with a larger weight, so the final system would prefer sentence structures mostly found in the desired type of text.

## 5. Conclusions

The experiments and the obtained results described in this manuscript show that using additional morphological knowledge for language model training can solve a large part of problems for highly inflective languages, as the Serbian language is. The proposed method incorporated the additional data into the words themselves, and one experiment used additional RNNLM word features on top of that. Big improvements were obtained both in *n*-gram systems and in RNNLM-based systems in relation to baseline systems which did not use any morphological data. The used Kaldi-RNNLM toolkit has also proven to be superior to any other previously used language model training toolkit for Serbian. There is still room for improvement, and there are future plans to create even better both acoustic and language models and even to further optimize the usage of morphological category information in the modeling of the Serbian language. Finally, an open-vocabulary language model capable of learning new words needs to be considered as well.

## Figures and Tables

**Table 1:** Morphological categories for words in the Serbian language.

Morphological category	Possible category values
Word type	Noun, pronoun, adjective, numeral, verb (variable), preposition, adverb, conjunction, particle, exclamation (invariable)
Word case	Nominative, genitive, dative, accusative, vocative, instrumental, locative
Grammatical number	Singular, plural
Grammatical gender	Masculine, feminine, neuter

**Table 2:** Audio database overview.

Database part	Amount of data (h)	Amount of speech (h)	Male (sub) speakers	Female (sub) speakers
Audio books	168	140	208	190
Radio talk shows	179	150	350	70
Phone recordings	61	41	169	181

Total	408	331	727	441
For training	379	308	677	410

**Table 3:** Textual database overview.

Corpus part	#Sentences	#Words	#Characters
Journalistic	737k	17M	94M
Literary	303k	3.9M	18M
Scientific	23k	503k	3M
Administrative	15k	378k	2M
Popular-scientific	18k	357k	2M
Conversational	38k	128k	530k
Transcriptions	251k	3.2M	15M

Total	1.4M	26M	135M
“Dev” set	20k	470k	2.6M

**Table 4:** Example of a feature vector in RNNLM experiments for word *sam* (Eng. *am*).

Index	Feature type	Feature	Value	Remarks
0	Constant	Constant	0.01	For math reasons
1–5	Special	Special word feat.	0	For bos/eos/unk/brk/silence
6	Unigram	Unigram prob.	0.00788	Scaled unigram log-prob.
7	Length	Word length	0.00186	Scaled word length
8–36	Word	1-hot vect. elem.	0	—
37	Word	1-hot vect. elem.	0.21	Scale based on unigram prob.
38–97644	Word	1-hot vect. elem.	0	—
97645–97657	Final	Lett. *n*-gram prob.	0	—
97658	Final	3-gram *-am$* pr.	0.12	Scaled letter 3-gram prob.
97659–97758	Final	Lett. *n*-gram prob.	0	—
97759	Final	2-gram *-m$* pr.	0.047	Scaled letter 2-gram prob.
97760–97869	Final	Lett. *n*-gram prob.	0	—
97870–98050	Initial	Lett. *n*-gram prob.	0	—
98051	Initial	2-gram *^s-* pr.	0.03	Scaled letter 2-gram prob.
98052	Initial	3-gram *^sa-* pr.	0.069	Scaled letter 3-gram prob.
98053–98144	Initial	Lett. *n*-gram prob.	0	—
98145–98300	Match	Lett. *n*-gram prob.	0	—
98301	Match	2-gram *-am-* pr.	0.064	Scaled letter 2-gram prob.
98302–100451	Match	Lett. *n*-gram prob.	0	—
100452	Match	2-gram *-sa-* pr.	0.057	Scaled letter 2-gram prob.
100453–100459	Match	Lett. *n*-gram prob.	0	—
100460	Match	3-gram *-sam-* pr.	0.11	Scaled letter 3-gram prob.
100461–101306	Match	Lett. *n*-gram prob.	0	—

**Table 5:** Some of the most frequent words, with and without morphology-based suffixes.

Without POS data	With POS data	Explanation
je	je_gl	*gl*agol = verb
i	i_vez	*vez*nik = conjunction
u	da_vez	—
da	u_pred_dat	*pred*log = preposition
se	se_zam	*zam*enica = pronoun
na	na_pred_dat	*dat*ive/locative
koji	koji_zam_nom_jd_mr	*nom*inative
bi	bi_gl_jd	*j*e*d*nina = singular
Srbije	Srbije_im_gen_jd_mr	*im*enica = noun, *gen*itive, *m*uški *r*od = masculine

**Table 6:** WER and CER results for 3-gram experiments, without and with additional POS data taken into account. Breakdown by test database part is shown as well.

Result	Total (%)	Books (%)	Shows (%)	“Mobile” (%)
WER 3-gram	8.89	6.25	12.64	**0.96**
WER 3-gram + POS	**6.90**	**4.12**	**10.45**	1.06

CER 3-gram	2.63	1.45	4.11	**0.40**
CER 3-gram + POS	**2.20**	**1.05**	**3.59**	0.42

**Table 7:** Lists of some of the most frequent word errors by type, with #occurrences (3-gram LM).

Substitutions without POS	Substitutions with POS	Insertions without POS	Insertions with POS	Deletions without POS	Deletions with POS
je ⟶ i (88)	je ⟶ i (79)	i (271)	je (242)	je (769)	je (742)
i ⟶ je (61)	i ⟶ je (50)	je (260)	i (235)	i (713)	i (669)
iz ⟶ i (48)	iz ⟶ i (39)	u (112)	u (88)	u (332)	u (302)
reko ⟶ rekao (42)	koji ⟶ koju (36)	da (87)	da (85)	da (215)	da (204)
koji ⟶ koju (40)	reko ⟶ rekao (32)	a (69)	a (54)	a (129)	a (130)
koja ⟶ koje (39)	sa ⟶ s (29)	na (54)	na (37)	on (121)	on (114)
koju ⟶ koje (37)	se ⟶ su (28)	po (31)	on (25)	na (99)	na (82)
sa ⟶ s (33)	je ⟶ oni (27)	o (28)	se (24)	to (76)	to (75)
nači ⟶ znači (31)	koji ⟶ koje (25)	ne (25)	o (22)	ja (75)	ja (63)
se ⟶ su (31)	nači ⟶ znači (25)	se (25)	pa (19)	od (63)	se (60)
je ⟶ koje (30)	koja ⟶ koje (24)	on (23)	od (17)	ne (62)	od (56)
tu ⟶ to (28)	mi ⟶ i (23)	—	—	se (61)	mi (54)
—	—	s (11)	ne (14)	—	—
kada ⟶ kad (22)	kada ⟶ kad (19)	kaže (10)	s (11)	joj (29)	sam (29)
imo ⟶ imao (19)	imo ⟶ imao (18)	koje (10)	kaže (10)	sam (28)	koji (25)
bilo ⟶ bila (19)	bila ⟶ bilo (18)	koji (10)	ovo (9)	koji (25)	joj (23)

**Table 8:** WER and CER results for RNNLM experiments, without and with additional POS data taken into account. Breakdown by test database part is shown as well.

Result	Total (%)	Books (%)	Shows (%)	“Mobile” (%)
WER RNNLM	4.90	2.77	7.56	**0.73**
WER RNNLM + POS	**4.34**	**2.18**	**6.93**	0.81

CER RNNLM	1.61	0.70	2.69	**0.28**
CER RNNLM + POS	**1.48**	**0.59**	**2.52**	0.33

## Data Availability

The audio and textual databases used to support the findings presented in this manuscript are partly available online and partly collected and owned by the Faculty of Technical Sciences in Novi Sad and the AlfaNum Company. All the mentioned data can be made available by the corresponding author upon request.
